# Maternal and Pregnancy Related Predictors of Cardiometabolic Traits in Newborns

**DOI:** 10.1371/journal.pone.0055815

**Published:** 2013-02-13

**Authors:** Katherine M. Morrison, Sonia S. Anand, Salim Yusuf, Stephanie A. Atkinson, Karleen M. Schulze, Purnima Rao-Melacini, Matthew J. McQueen, Sarah McDonald, Richard Persadie, Barry Hunter, Jacqueline Bourgeois, Jan W. Jansen, Koon K. Teo, the FAMILY (Family Atherosclerosis Monitoring In earLY Life) Study investigators

**Affiliations:** 1 Department of Pediatrics, Hamilton Health Sciences and McMaster University, Hamilton, Ontario, Canada; 2 Population Health Research Institute, Hamilton Health Sciences and McMaster University, Hamilton, Ontario, Canada; 3 Department of Medicine, McMaster University, Hamilton, Ontario, Canada; 4 Department of Clinical Epidemiology, McMaster University, Hamilton, Ontario, Canada; 5 Department of Obstetrics and Gynecology, McMaster University, Hamilton, Ontario, Canada; 6 Joseph Brant Hospital, Burlington, Ontario, Canada; 7 Department of Pathology and Molecular Medicine, McMaster University, Hamilton, Ontario, Canada; Medical University Innsbruck, Austria

## Abstract

**Background:**

The influence of multiple maternal and pregnancy characteristics on offspring cardiometabolic traits at birth is not well understood and was evaluated in this study.

**Methods and Findings:**

The Family Atherosclerosis Monitoring In earLY life (FAMILY) Study prospectively evaluated 11 cardiometabolic traits in 901 babies born to 857 mothers. The influence of maternal age, health (pre-pregnancy weight, blood pressure, glycemic status, lipids), health behaviors (diet, activity, smoking) and pregnancy characteristics (gestational age at birth, gestational weight gain and placental-fetal ratio) were examined. Greater gestational age influenced multiple newborn cardiometabolic traits including cord blood lipids, glucose and insulin, body fat and blood pressure. In a subset of 442 singleton mother/infant pairs, principal component analysis grouped 11 newborn cardiometabolic traits into 5 components (anthropometry/insulin, 2 lipid components, blood pressure and glycemia), accounting for 74% of the variance of the 11 outcome variables. Determinants of these components, corrected for sex and gestational age, were examined. Baby anthropometry/insulin was independently predicted by higher maternal pre-pregnancy weight (standardized estimate 0.30) and gestational weight gain (0.30; both p<0.0001) and was inversely related to smoking during pregnancy (−0.144; p = 0.01) and maternal polyunsaturated to saturated fat intake (−0.135;p = 0.01). Component 2 (HDL-C/Apo Apolipoprotein1) was inversely associated with maternal age. Component 3 (blood pressure) was not clustered with any other newborn cardiometabolic trait and no associations with maternal pregnancy characteristics were identified. Component 4 (triglycerides) was positively associated with maternal hypertension and triglycerides, and inversely associated with maternal HDL and age. Component 5 (glycemia) was inversely associated with placental/fetal ratio (−0.141; p = 0.005). LDL-C was a bridging variable between the lipid factors and glycemia.

**Conclusions:**

Maternal health, health behaviours and placenta to fetal weight ratio are associated with newborn cardiometabolic traits over and above gestational age. Future investigations are needed to determine if these factors remain important determinants of cardiometabolic health throughout childhood.

## Introduction

Cardiovascular (CV) disease is the leading cause of morbidity and mortality worldwide among adults and 9 modifiable risk factors account for the majority of this risk [1]. Four of these risk factors (tobacco use, dyslipidemia, elevated glucose and high blood pressure) are also associated with the majority of risk for stroke [2].These risk factors are also associated with atherosclerosis in youth [3], yet the primary determinants of these factors in young children are not well elucidated. Some evidence suggests that newborn adiposity, lipids, glycemia and blood pressure are influenced by maternal characteristics and intrauterine exposures [4], but most studies have examined each cardiometabolic trait separately.

We conducted a prospective investigation designed to understand determinants of cardiometabolic traits early in life in the Family Atherosclerosis Monitoring In earLY life (FAMILY) study, a longitudinal cohort study in which mothers were enrolled during pregnancy [5]. In this report, we sought to characterize newborn cardiometabolic characteristics, determine how they are interrelated and to investigate the influence of maternal factors on newborn cardiometabolic trait clusters.

## Methods

### Ethics Statement

The study was approved by the Research Ethics Boards at the participating hospitals (Hamilton Health Sciences, St Joseph’s Hospital – Hamilton, Joseph Brant Memorial Hospital, Burlington, ON).

The study rationale and design have been previously published [5]; 857 families consented and were enrolled during the mother’s pregnancy, drawing from three hospitals in Hamilton and Burlington, Ontario, Canada. In this report, data from the 901 index babies at birth and 857 mothers were included in the analysis.

### Maternal Characteristics

Maternal demographic, pregnancy history, laboratory and physical measurements were obtained from the mother at the baseline visit which occurred between 21 and 39 (median 28.6) weeks of gestation and chart review after delivery. Maternal height, weight, fasting lipid profile and glycemic status were measured. Maternal glycemic status was assessed using self-reported history of diabetes that preceded the pregnancy and results of a 75 g oral glucose tolerance test (OGTT) carried out on the non-diabetic pregnant mothers including fasting, 1 hour and 2 hour plasma glucose levels. Dysglycemia included pre-existing diabetes, impaired glucose tolerance (IGT) of pregnancy or gestational diabetes. Gestational diabetes was present when 2 or more plasma glucose values were equal to or exceeded the thresholds of: fasting 5.3 mmol/L, 1 hour 10.6 mmol/L, 2 hour 8.9 mmol/L set out by the Canadian Diabetes Association, and participants were classified with IGT of pregnancy when one threshold was exceeded [6]. Maternal pre-pregnancy weight and health during pregnancy were reported by the mothers and re-confirmed through chart review after delivery. Weight gain during pregnancy was derived from the difference between maternal recall of pre-pregnancy weight and the last clinic visit weight from the chart, prior to delivery.

Socioeconomic status was reported as annual household income. Family history of CV disease and diabetes and maternal health behaviours (cigarette smoking, dietary intake [7] and physical activity) were collected by self-report using validated questionnaires [1], [8]. Maternal nutritional intake was evaluated in mid-pregnancy utilizing a validated semi-quantitative food frequency questionnaire as previously described [9]. Nutritional variables considered in this analysis included total energy intake, macronutrient intake (% calories from carbohydrates, protein and fat) and energy adjusted intake of carbohydrate, protein, total fat, saturated (S), polyunsaturated fat (P), monounsaturated fat and trans fat. Nutrient composition was calculated as previously described [10], excluding records where the FFQ was >50% incomplete, or with implausible dietary intakes (<500 or >4500 kcal/d).Physical activity was self-reported utilizing a validated questionnaire [11]. Participants were categorized based on both work and leisure activities. “Mainly sedentary” indicated sedentary at work (or do not work) and during leisure (referent group), “Mildly active” indicating mild exercise during leisure or doing sedentary work, “Moderately active” if moderate exercise at leisure or walking plus climbing or lifting at work, “Highly active” if moderate exercise or heavy physical labour and “Extremely active” if strenuous activity in at least one of work or leisure with moderate activity in the other. (Table 1).

**Table 1:** Demographic characteristics and biochemical measures (mean and SD) for pregnant mothers at initial visit and after delivery.

Characteristics	Number of Observations	N (%) or Mean (SD)
Age (years)	857	32.1 (5.2)
Pre-pregnancy weight (kg)	728	72.3 (17.9)
Gestational weight gain (kg)	717	14.2 (5.6)
Height (cm)	854	164.4 (6.6)
LDL cholesterol (mmol/L)	828	3.49 (1.16)
HDL cholesterol (mmol/L)	854	1.93 (0.44)
Triglycerides (mmol/L)	854	2.39 (0.94)
Fasting glucose (mmol/L)-initial visit	854	4.47 (0.75)
Hb A1c		
At initial visit	854	0.052 (0.004)
At delivery	692	0.055 (0.005)
Glycemic status, n (%)	857	
Diabetic before pregnancy		35 (4.1)
Gestational diabetes		35 (4.1)
IGT		41 (4.8)
NGT		746 (87.1)
Systolic BP (mm Hg)	852	112.9 (9.7)
Diastolic BP (mm Hg)	852	70.3(7.9)
Blood Pressure Status, n (%)	853	
Elevated BP(HT, GHT, pre-eclampsia)		61 (7.1)
Normotensive		792 (92.9)
Medical History, n (%)		
CVD	853	3 (0.4)
Diabetes	803	35 (4.4)
Hypertension	853	35 (4.1)
Family history of CVD	853	168 (19.7)
Smoking history, n (%)	828	
Never smoked		515 (62.2)
Former smoker (prior to pregnancy)		196 (23.7)
Smoked anytime during pregnancy		117 (14.1)
Household Income <$30,000 n (%)	829	90 (10.9)
Physical activity during pregnancy, n (%)	853	
Mainly sedentary		132 (15.5)
Mildly active		247 (29.0)
Moderately active		277 (32.5)
Highly active		133 (15.6)
Extremely active		64 (7.5)
Total Energy (kCal/day)	765	2156 (749)
Daily intakes, % of energy		
Carbohydrates*		54.6 (6.3)
Protein		16.7 (2.6)
Total Fat**		29.2 (4.9)
Saturated Fat		11.0 (2.5)
Polyunsaturated Fat		4.0 (1.0)
Monounsaturated Fat		10.7 (2.1)
Trans Fat		0.2 (0.2)
Polyunsaturated:Saturated Fat Ratio		0.36 (0.1)

IGT: Impaired glucose intolerance; NGT: normal glucose tolerance; HT: pre-existing hypertension; GHT: gestational hypertension.

* After removing insoluble fibre.

** Note that the sum of fatty acid classes may not add up to the total fat, since total fat as analyzed may include some non-fatty acid material, such as, glycerol, phosphate, bound sugar or sterol analyzed in that food.

### Newborn Characteristics and Cardiometabolic Traits

Newborn characteristics including sex, gestational age at birth, and the placental:fetal weight ratio were recorded. The latter was calculated as: weight of the trimmed placenta (g)/birth weight (g). Newborn outcomes of interest included anthropometric characteristics (length, birth weight, body fat), blood pressure, glycemia (cord blood glucose), cord blood insulin and lipid profile (Table 2). Newborn length was measured on a length board and percent body fat was derived from measures of triceps and subscapular skin fold thickness (Lange callipers, Beta Technology Ltd, Cambridge, MD) measured at the birth visit [12], [13]. Blood pressure was measured with a Dinamap Pro100 V2 (GE Medical Systems, Tampa, Florida, USA) which utilizes an oscillometric method, and repeated 3 times at 2 minute intervals while the baby was sleeping or lying quietly. Umbilical venous cord blood, for analyses of the newborn’s lipids, glucose, insulin and apolipoprotein A1 (ApoA1) level, was collected in the Labour and Delivery unit immediately following birth. The blood samples were initially stored at 4°C, processed within 15 hours and stored long term at −165°C in liquid nitrogen. Total cholesterol (TC), high density lipoprotein cholesterol (HDL-C) and triglycerides (TG) were measured using enzymatic methods on the ROCHE INTEGRA analyzer, and low density lipoprotein cholesterol (LDL-C) was calculated using the Friedewald equation [14]. Glucose was measured utilizing a hexokinase method and insulin assayed by a manual radioimmunoassay process (Diagnostic Products Corp, Los Angeles, CA, USA). ApoA1 lipoprotein levels were assayed using immunoturbidometric methods (Roche/Hitachi 917 analyser with Tina-quant ApoA1 version 2 kits; Roche Diagnostics, Mannheim, Germany).

**Table 2:** Demographic characteristics and biochemical measures (mean and SD) for newborns.

Characteristics	Number of Observations	N (%) or Mean (SD)
Sex, n(%)	901	
Male		454 (50.4)
Female		447 (49.6)
Singletons n (%)	901	816 (90.6)
Multiple births		85 (9.4)
Gestational age (weeks) n (%)	901	38.9 (2.2)
<37 weeks		98 (10.9)
37 to <39 weeks		306 (34.0)
39 to <41 weeks		375 (41.6)
41+ weeks		122 (13.5)
Birth weight (kg)	900	3.36 (0.64)
Birth weight distribution, n (%):		
SGA (<10 percentile)		67 (7.4)
AGA (10 to 90 percentile)		713 (79.2)
LGA (>90 percentile)		120 (13.3)
Birth length (cm)	833	49.6 (2.9)
Percent body fat (%)	822	9.8 (2.7)
LDL-cholesterol (mmol/L)	683	0.72 (0.29)
HDL-cholesterol (mmol/L)	682	0.81 (0.29)
ApoA1 (g/L)	603	0.80 (0.16)
Triglyceride (mmol/L)	682	0.37 (0.21)
Glucose (mmol/L)	682	4.27 (0.95)
Insulin (pmol/L)	587	59.7 (124.7)
Systolic BP (mm Hg)	761	69.3 (9.6)
Diastolic BP (mm Hg)	761	39.1 (7.9)
Birth visit (days after birth)	865	2.6 (3.5)

### Statistical Analysis

Statistical analysis was conducted using SAS version 9.1 for unix, SAS Institute Inc., Cary, NC, USA. The distribution of all continuous variables was examined. Insulin and triglyceride were not normally distributed and were log transformed for further analysis. The impact of gestational age on the key newborn cardiometabolic traits (percent body fat, lipids, glucose levels, and blood pressure) was assessed using linear regression. Principal components analysis was used in a subset of mother/infant pairs to group the following potentially correlated newborn variables: birthweight, length, body fat, cord blood glucose and insulin, LDL-C, HDL-C, triglyceride, apoA1 and newborn systolic and diastolic blood pressure at the birth visit, into a smaller set of uncorrelated variables. Only data from singletons were included in the principal component analysis because multiple births influence birthweight, gestation at delivery, LDL-C, triglyceride and cord blood glucose levels and these multiple births shared common maternal environments. Prior to inclusion in the principal component analysis, each variable was adjusted for gestational age and sex, as these were strongly associated with birthweight and length, lipids, glucose, insulin and ApoA1 and blood pressure levels (Table 3). Blood pressure and body fat were further adjusted for the newborn’s age at birth visit (Tables 4, 5). These adjustments were carried out to improve the covariance structure and gestational age, sex and newborn age at birth visit were not included as potential determinants in the subsequent analyses.

**Table 3:** Newborn cardiometablic traits by gestational age at birth [mean (SE)].

	Gestational age at birth (weeks)				
	<37	37– <39	39– <41	41+	
	(n = 98)	(n = 306)	(n = 375)	(n = 122)	P-value
**LDL-C (mmol/L)**	0.95 (0.04)	0.72 (0.02)	0.68 (0.02)	0.69 (0.03)	<0.0001
**HDL-C (mmol/L)**	1.03 (0.04)	0.83 (0.02)	0.76 (0.02)	0.76 (0.03)	<0.0001
**ApoA1 (g/L)**	0.80 (0.02)	0.80 (0.01)	0.80 (0.01)	0.81 (0.02)	0.9874
**Triglyceride (mmol/L)**	0.23 (0.03)	0.30 (0.01)	0.40 (0.01)	0.52 (0.02)	<0.0001
**Glucose (mmol/L)**	3.92 (0.12)	4.10 (0.06)	4.39 (0.06)	4.54 (0.10)	<0.0001
**Insulin (pmol/L)**	108.6 (17.1)	71.8 (8.5)	46.6 (7.9)	36.0 (13.9)	0.0013
**Systolic BP (mmHg)**	67.0 (1.0)	69.5 (0.6)	69.2 (0.6)	71.2 (0.9)	0.0266
**Diastolic BP (mmHg)**	38.4 (0.8)	38.7 (0.5)	39.1 (0.4)	40.6 (0.8)	0.1616
**Percent Body fat (%)**	8.3 (0.4)	9.7 (0.2)	10.1 (0.1)	10.0 (0.2)	0.0002
**Birthweight (kg)**	2.29 (0.05)	3.27 (0.03)	3.59 (0.03)	3.72 (0.05)	<0.0001
**Birth Length (cm)**	44.7 (0.26)	49.0 (0.14)	50.6 (0.12)	51.2 (0.22)	<0.0001

**Table 4:** Blood pressure in newborns by age at time of measurement during birth visit [mean (SE)], adjusting for gestational age at birth.

	Age at Birth Visit (days)				
	0	1	2	>/ = 3	
	(n = 73)	(n = 370)	(n = 191)	(n = 231)	P-value
**Systolic BP (mmHg)**	64.8 (1.1)	66.9 (0.5)	70.5 (0.7)	75.1 (0.8)	<0.0001
**Diastolic BP (mmHg)**	35.6 (0.9)	37.3 (0.4)	40.1 (0.6)	43.1 (0.6)	<0.0001
**Body Fat (%)**	9.7 (0.31)	9.7 (0.14)	9.5 (0.19)	10.2 (0.19)	0.099

**Table 5:** Standardized Estimates in univariate analysis of key newborndeterminants, adjusted for gestational age of newborn at birth.

	*LDL-C*	*HDL-C*	*TG*	*Glucose*	*Systolic BP*	*Diastolic BP*	*% Body Fat*
**Singletons**	−**0.230** ***	−0.071	−**0.112** **	**0.199** ***	0.024	0.014	**0.147** ***
**Females vs males**	**0.105** **	**0.101** **	−0.018	0.030	0.028	0.040	0.066
**Placental-fetal ratio**	**0.099** *	**0.144** **	−0.009	−**0.125** **	−0.016	−0.039	0.019
**Age at birth visit**					0.286****	**0.257** ***	**0.168** ***

* p<0.05;

** p<0.01;

*** p<0.0001.

Principal component analysis utilizes the linear relationship among variables to reduce the number of variables to summary factors or components while retaining as much of the total variance in the original data as possible [15]. Individual variables “load” onto a component if they are highly correlated with the summary component. We used an eigenvalue (sum of the squared factor loadings) of >1 criterion to retain components [15]. Varimax rotation, a linear transformation, was used to maximize the variance explained by each factor. In interpreting the rotated components, an observed variable with loading value greater than 0.3 was used to interpret information conveyed by the variables contributed to each rotated component.

Using the resulting 5 newborn principal components as dependent variables, we then examined associations with each maternal characteristic using simple linear regression. We considered maternal age, pre-pregnancy weight, gestational weight gain, glucose intolerance, mid-pregnancy lipid profile (HDL-C, LDL-C, triglycerides), smoking during the pregnancy, elevated blood pressure prior to or during pregnancy, placental-fetal weight ratio, major macronutrient intake (carbohydrate, protein, fat and P:S fat ratio), physical activity during pregnancy, maternal education, and annual household income as independent variables. These variables were selected based on prior reports of association in the medical literature and our beliefs regarding possible pathophysiological mechanisms linking maternal characteristics to newborn cardiometabolic traits. All factors which were associated with a newborn component with p<0.10 were entered into a multivariate linear regression model which included maternal age.

## Results

### Participants

We enrolled 857 families between 2002 and 2009, with 857 mothers and 901 babies (816 singleton and 85 twins or triplets). Most mothers (92.3%) were married, of European ethnicity (84.6%) and had attended college or university (83.9%). Approximately 11% reported a family income<$30,000 CDN annually (the Canadian poverty cut-off level) and 35% had a household income that exceeded $100,000 CDN annually. While 37.8% of mothers were current or former smokers, 14.1% reported smoking for some time during the pregnancy. Hypertension predating the pregnancy was reported in 4.1% and a very small percentage (0.7%) developed pre-clampsia during pregnancy. Abnormal glycemic status (dysglycemia) was present in 111 (13.0%) mothers during the pregnancy; 35 (4.1%) had pre-existing diabetes, 35 (4.1%) were diagnosed with gestational diabetes and 41 (4.8%) had impaired glucose tolerance of pregnancy. A family history of CV disease (angina, myocardial infarction or stroke) was reported by 19.7% of mothers. Dietary records were available for 765 mothers. Maternal energy intake averaged 2156±749 kcal/day and reported carbohydrate and protein intakes met current guidelines, whereas the consumption of saturated fat was high relative to polyunsaturated fat intake (P:S ratio = 0.36). (Table 1).

### Characteristics of Newborns

There were 901 live births and no stillbirths; two infants died at 1 and 2 days of age; 454 (50.4%) were male and 377(41.8%) were first babies for the mothers. The mean gestational age at birth was 38.9 (2.2) weeks; 98 (10.9%) were born prior to 37 weeks gestation (3). Cord blood was successfully collected in 683 (75.8%) of the births and lipid profile, glucose and insulin levels were measured in these samples. In 218 births, cord blood was not available for one or more of several reasons including birth outside of hospital, family participation in cord blood banking, precipitous or complicated delivery or failure of the labour and delivery unit staff to note that the family was participating in the study. Weight was taken at birth and birth length, skinfolds and blood pressure were measured at the birth visits on average 2.6 (3.5) days after birth. Blood pressure measurements could not be made in 110 (12.2%) babies because they were restless or crying or in 25 (2.9%) who had no birth visit due to early discharge.

### Association of Cord Blood Lipids, Glucose, Percent Body Fat and BP with Gestational Age

Newborn levels of LDL-C, HDL-C (but not ApoA1) and insulin were lower with more advanced gestational age, whereas glucose and triglycerides were higher (all p<0.001). Similarly, percent body fat (p = 0.0002) and systolic BP (p = 0.03), but not diastolic BP, were higher with increasing gestational age (Table 3).Both systolic and diastolic blood pressures were significantly higher with increasing age (days) at the time of measurement, after adjusting for gestational age at birth (Table 4).

Table 5 shows some of the variables which significantly influenced the newborns’ cardiometabolic profile at birth, after adjusting for gestational age. These include singletons compared to multiple births, female compared to male children and the age in days for the first visit after birth.

### Principal Component Analysis

Principal component analysis was conducted in a subset of 442 singletons for whom all 11 outcome variables of interest were available. There were no differences in maternal characteristics between those who were included and those who were not, except for the newborn characteristics that were related to exclusion of twins and triplets (gestational age at birth, length, birthweight, LDL-C and glucose). (Table S1).

As shown in Table 6, the first five components displayed eigenvalues greater than 1.0, thus meeting our criteria for acceptance of a component; collectively they accounted for 74% of the total variance for the 11 variables. Table 7 shows the variable “loadings” or contributions for both the unrotated and the rotated principal components. Rotation of components made them cleaner for interpretation and reduced the number of bridge variables (variables which load on 2 or more summary components). The first component, anthropometry/insulin, is loaded by birthweight, percent body fat, birth length, and insulin; birthweight had the highest factor loading. The newborn HDL-C/ApoA1 component (component 2), is loaded by ApoA1, HDL-C and LDL-C, with HDL-C and ApoA1 having higher factor loading than LDL-C. In the newborn blood pressure component (component 3), both systolic and diastolic BP had equal factor loadings. The second newborn lipid component, triglycerides (component 4), triglycerides had higher factor loading than LDL-C and HDL-C was not related. Glucose and LDL-C loaded on the newborn glycemia component (component 5) and glucose had higher factor loading (Table 7).

**Table 6:** Eigenvalues and their proportion of explained variance from Principal component analysis.

Component	Eigenvalue	Proportion of Variance	Cumulative Variance
**1**	**2.52**	**0.23**	**0.23**
**2**	**1.86**	**0.17**	**0.40**
**3**	**1.58**	**0.14**	**0.54**
**4**	**1.15**	**0.11**	**0.65**
**5**	**1.02**	**0.09**	**0.74**
6	0.92	0.08	0.82
7	0.62	0.06	0.88
8	0.57	0.05	0.93
9	0.35	0.03	0.96
10	0.22	0.02	0.98
11	0.17	0.02	1.00

Eleven newborn characteristics were included in a principal component analysis. Five components with an eigenvalue >1 were kept for further analysis.

**Table 7:** Variable Loadings for the First 5 Principal Components.

	Unrotated Principal Components					Rotated Principal Components				
	PC 1	PC 2	PC 3	PC 4	PC 5	PC 1	PC 2	PC 3	PC 4	PC 5
Birth weight	**.88**	−.13	−.19	.02	.16	**.92**	.00	.02	−.06	−.05
Birth length	**.73**	−.11	−.21	.09	.07	**.77**	.02	−.04	.06	.05
Percent body fat	**.71**	−.14	−.13	.00	.21	**.76**	−.04	.03	.01	−.10
Log Insulin	**.60**	−.04	.02	.19	−.14	**.56**	.02	.17	−.15	.24
HDL cholesterol	.16	**.86**	−.18	−.16	−.22	.00	**.89**	.03	−.27	−.02
Apo A1	.04	**.85**	−.19	.19	−.01	−.02	**.87**	.02	.10	.17
LDL cholesterol	−.01	**.50**	−.13	−.11	**.64**	.06	**.50**	−.01	**.49**	−**0.43**
Diastolic BP	.21	.18	**.85**	.05	.03	−.01	0	**.90**	.00	.00
Systolic BP	**.34**	.21	**.81**	.04	.04	.12	.04	**.90**	−.02	**−**002
Log Triglycerides	−**.40**	−.08	.05	**.58**	**.59**	−**.**20	−.14	−.02	**.87**	.18
Glucose	.02	.09	−.10	**.83**	−**.34**	.05	.12	−.03	**.**14	**.89**

Variable loadings >0.3 are in bold to improve interpretability. The 5 components identified are labelled as follows: anthropometry/insulin (PC1), HDL-C/ApoA1 (PC2), Blood Pressure (PC3), Triglyceride (PC4) and Glycemia (PC5).

A bridging variable is identified when a newborn variable is correlated with more than one component. Newborn LDL-C bridged the two newborn lipid components (components 2 and 4) and newborn glycemia (component 5) and in each component had the minor role compared to the other newborn cardiometabolic traits. Table 7 illustrate these 5 components and their inter-relationships.

### Influence of Maternal Characteristics on Newborn Cardiometabolic Traits

**Figure 1:**
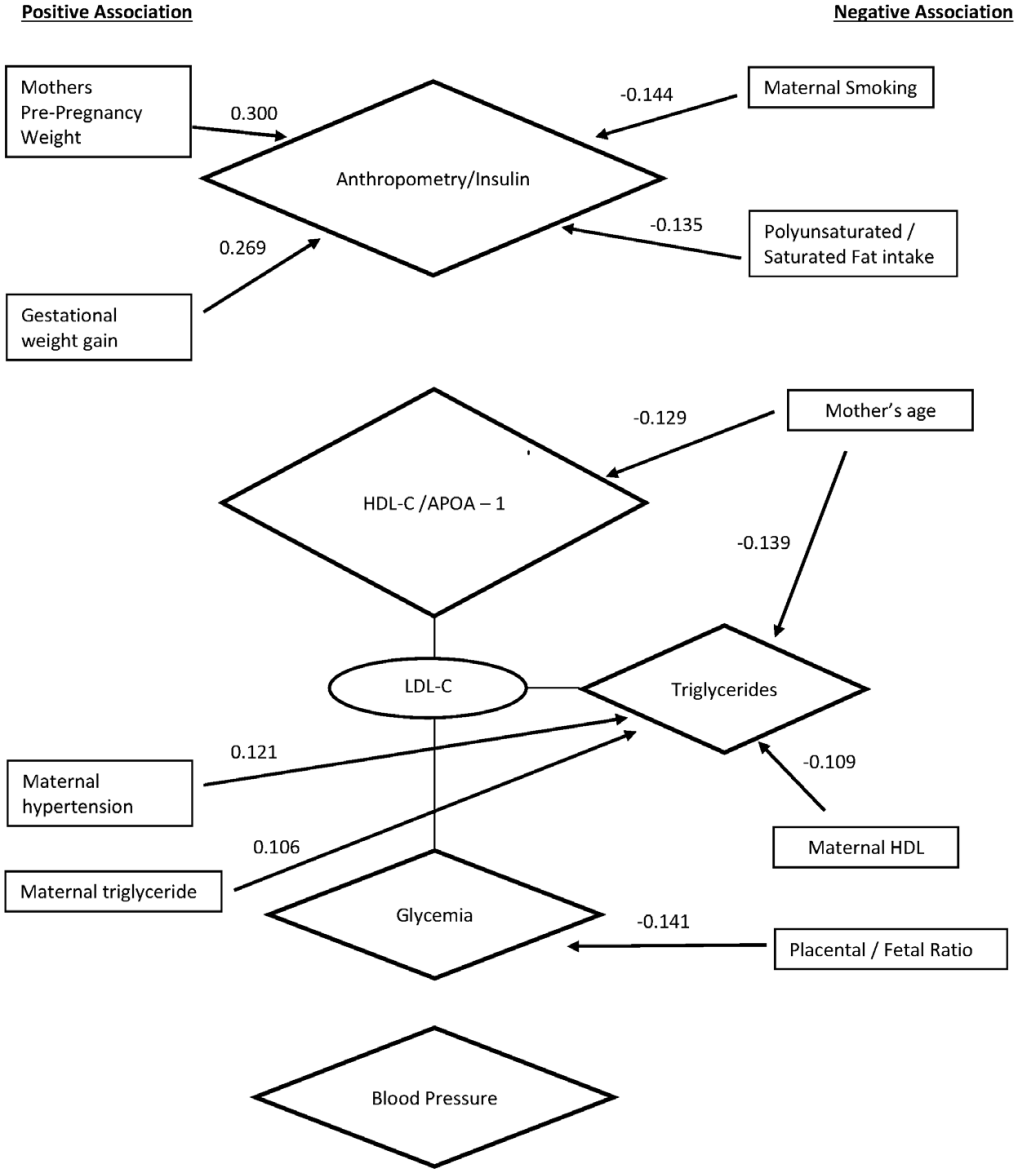
Visual representation of the relationship between newborn cardiometabolic components (illustrated with diamonds) and the maternal/pregnancy variables associated with them (in rectangles). Numbers beside the arrows represents the strength and direction of the relationship between the variable and the component. Variables within ovals “bridge” 2 components as they are loaded onto more than one component. All newborn traits were adjusted for gestational age and sex prior to principal component analysis. Blood pressure and body fat were also adjusted for age at time of measurement.

Maternal characteristics which were associated with each newborn component with p<0.10 and which were included in multivariable models are shown in Table 8. Maternal factors including pre-pregnancy weight (Standardized Estimate (SE): 0.30; p<0.0001), gestational weight gain (SE: 0.27; p<0.0001), maternal smoking during pregnancy (SE: −0.14; p = 0.007) and maternal P:S fat intake ratio(SE: −0.14; p = 0.009) were independently and significantly associated with Component 1: newborn anthropometry/insulin. Components 2, 4 and 5 all included Newborn LDL-C as a minor component. Thus, LDL-C was a bridging variable between the two lipid components and the newborn glycemia component. The newborn HDL-C-ApoA1 (Principal Component 2) was related to maternal age and maternal P:S fat intake ratio in univariate analysis, but only maternal age (SE: −0.13; p = 0.01) was independently related in multivariate analysis. Several maternal characteristics were independently, albeit weakly related to the newborn triglyceride component (Component 4). Maternal age (SE: −0.14; p = 0.005) and maternal HDL-C (SE: −0.11; p = 0.04) were inversely related and maternal hypertension (SE: 0.12; p = 0.02) and maternal triglycerides (SE: 0.11; p<0.05) were directly related. The component that explained the least of the variability of the newborn cardiometabolic traits was newborn glycemia (component 5) and the only characteristic that was related to this in multivariate analysis was the placental-fetal ratio (SE: −0.14; p = 0.005). Newborn blood pressure (Component 3) was not related to any maternal or pregnancy related characteristic in the multivariate analysis. (Table 8, Figure 1).

**Table 8:** Maternal variables as predictors of principal components (N = 442).

	Univariable Analyses		Multivariable Analyses	
Predictors	StandardizedParameter Estimate	P-value	StandardizedParameter Estimate	P-value
**Component #1: Newborn’s Anthropometry/Insulin**				
Maternal age (yr)	0.118	0.01	0.063	0.26
Maternal pre-pregnancy weight (kg)	0.257	<0.0001	0.300	<0.0001
Maternal gestational weight gain (kg)	0.126	0.01	0.269	<0.0001
Maternal glucose intolerance/dysglycemia	0.208	<0.0001	0.099	0.06
Maternal smoking during pregnancy	−0.139	0.004	−0.144	0.01
Household income <$30,000 CDN	−0.116	0.02	−0.107	0.05
Placental-fetal ratio	0.095	0.06	0.056	0.27
Maternal HDL-C level	−0.118	0.01	−0.094	0.09
Maternal energy adjusted carbohydrates	−0.087	0.07	−0.045	0.39
Maternal polyunsaturated: saturated fat	−0.109	0.03	−0.135	0.01
Log Maternal triglycerides	0.109	0.02	0.023	0.68
**Component # 2: Newborn’s HDL-C/ApoA1/LDL-C**				
Maternal age	−0.115	0.02	−0.129	0.01
Maternal pre-pregnancy weight (kg)	−0.087	0.08	−0.081	0.11
Maternal smoking during pregnancy	−0.080	0.09	−0.051	0.32
Maternal polyunsaturated: saturated fat	−0.093	0.06	−0.089	0.08
**Component # 3: Newborn’s Systolic/Diastolic BP**				
Maternal age (yrs)	0.035	0.46	0.028	0.56
Maternal glucose intolerance/dysglycemia	0.094	0.049	0.091	0.06
**Component # 4: Newborn’s Triglycerides/LDL-C**				
Maternal age	−0.055	0.25	−0.139	0.0053
Maternal pre-pregnancy weight (kg)	0.088	0.08	0.031	0.55
Maternal glucose intolerance/dysglycemia	0.094	0.047	0.023	0.66
Maternal elevated BP	0.115	0.02	0.121	0.02
Maternal HDL-C level	−0.135	0.005	−0.109	0.04
Log Maternal triglycerides	0.129	0.007	0.106	0.046
**Component # 5: Newborn’s Glycemia/LDL-C**				
Maternal age	0.023	0.63	−0.005	0.92
Placental-fetal ratio	−0.144	0.004	−0.141	0.005
Maternal HDL-C level	0.100	0.04	0.098	0.05

## Discussion

In this prospective birth cohort of primarily white Caucasian women from Ontario Canada, we identified 5 clusters of newborn cardiometabolic factors including anthropometrics, two lipid components, blood pressure and a glycemia component which collectively account for 74% of the variance of the newborn cardiometabolic traits. We then found that key maternal characteristics including maternal age, pre-pregnancy weight, pregnancy weight gain, the relative intake of polyunsaturated to saturated fat, hypertension, tobacco use, second trimester HDL-C, triglyceride and the placental to fetal weight ratio are significantly associated with these characteristics (Figure 1). Maternal physical activity, carbohydrate and protein intake and socioeconomic status were not identified as independent determinants of any of the newborn cardiometabolic clusters. To our knowledge our study is the first to identify clusters of cardiometabolic traits in newborns and to report the association of maternal and pregnancy related characteristics to these clusters [16]–[18]. This approach is useful to identify maternal influences on key newborn cardiometabolic traits that may be targeted in future intervention or mechanistic studies.

Gestational age is strongly associated with lower cord blood LDL-C, HDL-C and insulin and higher newborn triglycerides, glucose, body fat and systolic blood pressure. The influence of gestational age on newborn cardiometabolic factors has been previously documented but was not always considered in previous studies examining maternal influences on cardiometabolic outcomes at birth. Premature (gestational age <37 weeks) newborns have increased cardiovascular risk factors as adults [19]. After taking into account the profound effect of infant maturity (gestational age) on newborn cardiometabolic status, we investigated the interrelationship between newborn traits and maternal/pregnancy influences.

We show that newborn cardiometabolic traits group into 5 clusters. Of these, the newborn anthropometry/insulin cluster alone explains 23% of the variance in newborn cardiometabolic traits. Maternal pre-pregnancy weight and weight gain throughout pregnancy are strongly positively associated, whereas maternal smoking and relative consumption of polyunsaturated to saturated fat are negatively associated with newborn anthropometry/insulin. Maternal glucose status was strongly correlated to the anthropometric/insulin factor in univariate analysis (SE 0.21; p<0.0001), but only weakly related in multivariate analysis (SE: 0.10; p = 0.06) when the influence of maternal weight and pregnancy weight gain were included. While maternal weight and maternal smoking behaviours have been previously related to newborn body size, some findings are new, including the influence of maternal diet (P:S fat intake ratio) on offspring body size. Saturated fats in this study were mostly derived from consumption of dairy products, red meat and chocolate whereas polyunsaturated fats were mostly derived from peanut butter and chicken. The average P/S ratio in our study of 0.36 reflects a diet predominantly comprised of saturated fats, and is significantly lower than the Canadian average (0.55) [20] and the US recommendations for prevention of CV disease [21]. Maternal milk consumption has been associated with increased birthweight in 2 large prospective cohort studies [22], [23] although the association has been attributed to milk protein and not to saturated fat. In contrast, maternal protein intake was not related to newborn anthropometrics in univariate analysis. A recent randomized trial among women at risk for gestational diabetes compared intensive lifestyle changes to usual care with the primary goal being prevention of gestational diabetes. In this trial, women in the intervention group consumed increased polyunsaturated and reduced saturated fat and had newborns with lower birthweight for gestational age (3,532 g versus 3,659 g, p = 0.035) and a lower incidence of large for gestational age newborns 12.1% versus 19.7%, p = 0.042 than control [24]. These findings are consistent with our observation, and may in part explain birthweight variations between populations who consume large amounts of animal products high in saturated fat compared to those who do not [25].

Cord blood LDL-C levels bridged three components including 2 lipid components and the glycemia component. Together, the HDL-C/ApoA cluster and the newborn triglyceride cluster explain 28% of the variance in newborn cardiometabolic traits. We have demonstrated lower newborn HDL-C and LDL-C with advancing gestational age as previously reported, but no difference in ApoA1 was identified [26]. The maternal lipid profile is characterized by higher levels of LDL-C and HDL-C during pregnancy compared to non-pregnancy [27], [28] and had only weak influence on newborn cardiometabolic components. Maternal LDL-C was not related to any newborn cardiometabolic cluster and maternal HDL-C was weakly associated with lower newborn triglyceride. Both maternal triglyceride and hypertension were positively associated with newborn triglyceride. Elevated cord blood triglyceride levels have been seen in the cord blood of offspring exposed to adverse perinatal conditions including maternal hypertension [29], although the mechanism for this remains uncertain. Increased cord blood LDL-C and triglyceride have been described in one previous study of offspring of mothers with pre-eclampsia (n = 11), but these authors did not account for gestational age nor consider other maternal characteristics as potential determinants of the newborn lipid profile [30]. Maternal age was inversely associated with both the newborn triglyceride and HDL-C/Apo A1 components. Future studies investigating the link between maternal and newborn genetic factors on lipid levels at birth may further elucidate the determinants of newborn lipids.

Component 5, newborn glycemia, explained 9% of the variance in newborn cardiometabolic traits. The influence of placental function is demonstrated by the strong inverse association between placental-fetal weight ratio and newborn glycemia that is independent of maternal glucose intolerance. As the organ for nutrient transport and gas exchange between mother and fetus, the placenta is implicated in disturbances of fetal growth. Some studies suggest that the relative size of the placenta to birth weight is associated with antenatal factors including cigarette smoking, hypertension, and maternal weight gain [31]. The placental-fetal weight ratio has also been shown to be associated with the CV risk factors hypertension and impaired glucose tolerance, in childhood and adulthood [32]–[34]. In a contemporary Australian cohort [35], increased placental weight-birth weight ratio was associated with clustering of cardiometabolic risk factors at 8 years of age, although exposures to maternal tobacco, breastfeeding, and offspring weight gain after the first year of life were also identified as important influences. In contrast, in our multivariate analysis including a broad selection of maternal and pregnancy characteristics, the placental-fetal ratio was inversely related to newborn glycemia and was not related to other newborn cardiometabolic components. Maternal smoking has been linked to adverse metabolic outcomes in children and adolescents as recently reviewed [36], but in our study in newborns, it was only related to the anthropometry/insulin component.

The mean systolic blood pressure (69.3±9.6 mmHg) was within the normal range expected for sleeping newborns of a similar age and systolic blood pressure was directly related to gestational age at birth and time since delivery. This is consistent with previous studies [37] and likely reflects the dynamic changes in the vascular circulation that occur in the immediate postnatal period. After controlling for age, sex and newborn age at the time of blood pressure measurement, only maternal dysglycemia was related to newborn blood pressure in univariate analysis (SE:0.09; p = 0.05) and no maternal or pregnancy characteristics were related to newborn blood pressure in multivariate analysis. (Figure 1).While previous studies have shown that maternal age influences newborn blood pressure and we have also shown that maternal age influences the lipid components, we identified no influence on newborn blood pressure.

### Strengths and Limitations

Our study is the first to explore the clustering of cardiometabolic traits at birth using principal component analysis and to simultaneously examine the association between the derived components and maternal determinants in a large number of babies and their mothers. Previous studies typically reported multiple single factor associations without performing a unifying analysis [38], [39]. In addition, most prior studies have reported the association between maternal and pregnancy factors and cardiometabolic risk factors in older children [38], [39]. Limitations of our study include reliance on self-reported health behaviours which may have influenced our conclusions on the importance of these variables in influencing offspring health. However, all of our questionnaires have been previously validated and many of our findings have face validity and are supported by prior studies.

### Conclusion

Maternal health (age, pre-pregnancy weight, weight gain during pregnancy, HDL-C, triglyceride, hypertension), health behaviours (tobacco exposure, polyunsaturated/saturated fat intake) and placental to fetal weight ratio are important determinants of newborn cardiometabolic health over and above gestational age. Future investigations are required to determine if these variables remain important determinants of cardiometabolic health as the offspring grow.

## Supporting Information

Table S1 Maternal and Newborn Demographic Characteristics and Biochemical Measures for those included and not included in the Principal Component Analysis.
(DOCX)
