# Supplementary material for: Maternal and Pregnancy Related Predictors of Cardiometabolic Traits in Newborns
Source: PLoS One. 2013 Feb 13;8(2):e55815. doi: 10.1371/journal.pone.0055815 (PMC3572188; doi:10.1371/journal.pone.0055815)
Supplement: Table S1 — Maternal and Newborn Demographic Characteristics and Biochemical Measures for those included and not included in the Principal Component Analysis. (DOCX) [file pone.0055815.s001.docx]

Table S1. Maternal and Newborn measures for those included and not included in the Principal Component Analysis

|  | **All Subjects** | | **Subjects used in PC analyses** | | **Subjects not used in PC analyses** | |  |
| --- | --- | --- | --- | --- | --- | --- | --- |
| **Characteristics** | **Number of Observations** | **N (%) or Mean (SD)** | **Number of Observations** | **N (%) or Mean (SD)** | **Number of Observations** | **N (%) or Mean (SD)** | **p-value** |
| **Newborn Factors** |  |  |  |  |  |  |  |
| Sex, n(%) | 901 |  | 442 |  | 459 |  |  |
| Male |  | 454 (50.4) |  | 229 (51.8) |  | 225 (49.0) | 0.40 |
| Female |  | 447 (49.6) |  | 213 (48.2) |  | 234 (51.0) |  |
| Singletons n (%) | 901 | 816 (90.6) | 442 | 442 (100) | 459 | 85 (18.5) | <.0001 |
| Multiple births |  | 85 (9.4) |  | 0 (0) |  | 374 (81.5) |  |
| Gestational age (weeks) n (%) | 901 | 38.9 (2.2) | 442 | 39.3 (1.4) | 459 | 38.4 (2.6) | <.0001 |
| <37 weeks |  | 98 (10.9) |  | 20 (4.5) |  | 78 (17.0) | < 0001 |
| 37 to <39 weeks |  | 306 (34.0) |  | 155 (35.1) |  | 151 (32.9) |  |
| 39 to <41 weeks |  | 375 (41.6) |  | 201 (45.5) |  | 174 (37.9) |  |
| 41+ weeks |  | 122 (13.5) |  | 66 (14.9) |  | 56 (12.2) |  |
| Birth weight (kg) n (%) | 900 | 3.36 (0.64) | 442 | 3.52 (0.50) | 458 | 3.20 (0.72) | <.0001 |
| SGA (<10 percentile) |  | 67 (7.4) |  | 26 (5.9) |  | 41 (9.0) | .004 |
| AGA (10 to 90 percentile) |  | 713 (79.2) |  | 342 (77.4) |  | 371 (81.0) |  |
| LGA (>90 percentile) |  | 120 (13.3) |  | 74 (16.7) |  | 46 (10.0) |  |
| Birth length (cm) | 833 | 49.6 (2.9) | 442 | 50.2 (2.2) | 391 | 48.8 (3.4) | <.0001 |
| Percent body fat (%) | 822 | 9.8 (2.7) | 442 | 9.8 (2.5 ) | 380 | 9.8 (3.0) | 0.85 |
| LDL-cholesterol (mmol/L) | 683 | 0.72 (0.29) | 442 | 0.68 (0.27) | 241 | 0.78 (0.32) | <.0001 |
| HDL-cholesterol (mmol/L) | 682 | 0.81 (0.29) | 442 | 0.79 (0.27) | 240 | 0.84 (0.31) | 0.06 |
| ApoA1 (g/L) | 603 | 0.80 (0.16) | 442 | 0.79 (0.15) | 161 | 0.82 (0.17) | 0.10 |
| Triglyceride (mmol/L) | 682 | 0.37 (0.21) | 442 | 0.37 (0.22) | 240 | 0.36 (0.20) | 0.52 |
| Glucose (mmol/L) | 682 | 4.27 (0.95) | 442 | 4.36 (0.96) | 240 | 4.10 (0.92) | <.001 |
| Insulin (pmol/L) | 587 | 59.7 (124.7) | 442 | 61.2 (133.5) | 145 | 55.0 (93.2) | 0.53 |
| Systolic BP (mm Hg) | 761 | 69.3 (9.6) | 442 | 69.3 (9.8) | 319 | 69.4 (9.4) | 0.92 |
| Diastolic BP (mm Hg) | 761 | 39.1 (7.9) | 442 | 38.8 (7.8) | 319 | 39.4 (8.0) | 0.32 |
| Birth visit (days after birth) | 865 | 2.6 (3.5) | 442 | 1.6 (1.6) | 423 | 3.7 (4.4) | <.0001 |

| **Maternal Factors** |  |  |  |  |  |  |  |
| --- | --- | --- | --- | --- | --- | --- | --- |
| Age (years) | 857 | 32.1 (5.2) | 442 | 32.0 (5.4) | 415 | 32.1 (4.9) | 0.68 |
| Pre-pregnancy weight (kg) | 728 | 72.3 (17.9) | 402 | 73.8 (18.9) | 326 | 70.5 (16.4) | 0.01 |
| Gestational weight gain (kg) | 717 | 14.2 (5.6) | 397 | 14.4 (5.4) | 320 | 14.0 (5.9) | 0.42 |
| Height (cm) | 854 | 164.4 (6.6) | 442 | 164.4 (6.7) | 412 | 164.3 (6.6) | 0.79 |
| LDL cholesterol (mmol/L) | 828 | 3.49 (1.16) | 429 | 3.39 (1.13) | 399 | 3.60 (1.18) | 0.007 |
| HDL cholesterol (mmol/L) | 854 | 1.93 (0.44) | 439 | 1.95 (0.43) | 415 | 1.92 (0.45) | 0.41 |
| Triglycerides (mmol/L) | 854 | 2.39 (0.94) | 439 | 2.35 (0.86) | 415 | 2.43 (1.02) | 0.23 |
| Fasting glucose (mmol/L)-initial | 854 | 4.47 (0.75) | 440 | 4.49 (0.61) | 414 | 4.45 (0.87) | 0.52 |
| Hb A1c |  |  |  |  |  |  |  |
| At initial visit | 854 | 0.052 (0.004) | 439 | 0.052 (0.004) | 415 | 0.052 (0.005) | 0.79 |
| At delivery | 692 | 0.055 (0.005) | 436 | 0.055 (0.004) | 256 | 0.055 (0.005) | 0.57 |
| Glycemic status, n (%) | 857 |  | 442 |  | 415 |  |  |
| Diabetic before pregnancy |  | 35 (4.1) |  | 19 (4.3) |  | 16 (3.9) | 0.55 |
| Gestational diabetes |  | 35 (4.1) |  | 22 (5.0) |  | 19 (4.6) |  |
| IGT |  | 41 (4.8) |  | 22 (5.0) |  | 13 (3.1) |  |
| NGT |  | 746 (87.1) |  | 379 (85.8) |  | 367 (88.4) |  |
| Systolic BP (mm Hg) | 852 | 112.9 (9.7) | 440 | 113.8 (9.7) | 412 | 111.9 (9.5) | 0.005 |
| Diastolic BP (mm Hg) | 852 | 70.3 (7.9) | 440 | 70.6 (8.1) | 412 | 70.1 (8.1) | 0.35 |
| Medical History, n (%) |  |  |  |  |  |  |  |
| CVD | 853 | 3 (0.4) | 439 | 2 (0.5) | 414 | 1 (0.2) | 1.00 |
| Diabetes | 803 | 35 (4.4) | 439 | 19 (4.3) | 364 | 16 (4.4) | 0.96 |
| Hypertension | 853 | 35 (4.1) | 439 | 19 (4.3) | 414 | 16 (3.9) | 0.73 |
| Blood Pressure Status, n (%) | 853 |  | 439 |  | 414 |  |  |
| Elevated BP |  |  |  |  |  |  |  |
| (HT, GHT, pre-eclampsia) |  | 61 (7.1) |  | 34 (7.7) |  | 27 (6.5) | 0.49 |
| Normotensive |  | 792 (92.9) |  | 405 (92.3) |  | 387 (93.5) |  |
| Family history of CVD | 853 | 168 (19.7) | 439 | 82 (18.7) | 414 | 86 (20.8) | 0.44 |
| Smoking history, n (%) | 828 |  | 438 |  | 390 |  |  |
| Never smoked |  | 515 (62.2) |  | 255 (58.2) |  | 260 (66.7) | 0.04 |
| Former smoker (quit prior to pregnancy) |  | 196 (23.7) |  | 113 (25.8) |  | 83 (21.3) |  |
| Smoked during pregnancy |  | 117 (14.1) |  | 70 (16.0) |  | 47 (12.0) |  |
| Did not smoke in pregnancy |  | 711 (85.9) |  | 368 (84.0) |  | 343 (88.0) | 0.11 |
| Household Income, n (%) | 829 |  | 428 |  | 401 |  |  |
| <$30,000 |  | 90 (10.9) |  | 49 (11.4) |  | 41 (10.2) | 0.57 |
